# Silica Aerogel Hybridized with Melamine-Terephthalaldehyde Polymer for In-Tube Solid-Phase Microextraction of Polycyclic Aromatic Hydrocarbons from Environment Water

**DOI:** 10.3390/nano12101766

**Published:** 2022-05-22

**Authors:** Qiong Jiang, Shuwu Zhang, Juanjuan Feng, Min Sun

**Affiliations:** 1 College of Plant Protection, Gansu Agricultural University/Biocontrol Engineering Laboratory of Crop Diseases and Pests of Gansu Province, Lanzhou 730070, China; zhangsw704@126.com; 2 Key Laboratory of Interfacial Reaction & Sensing Analysis in Universities of Shandong, School of Chemistry and Chemical Engineering, University of Jinan, Jinan 250022, China; chm_fengjuanjuan@ujn.edu.cn

**Keywords:** solid-phase microextraction, silica aerogel, melamine-terephthalaldehyde polymer, polycyclic aromatic hydrocarbons, online analysis

## Abstract

To improve the extraction performance of the silica aerogel, a melamine-terephthalaldehyde polymer was used to hybridize silica aerogel, and the hybridized aerogel was coated on the surface of stainless steel wire to prepare a fiber-filled extraction tube through placing four wires into a polyetheretherketone tube. The tube was combined with high-performance liquid chromatography, then the online extraction and detection were established. Several polycyclic aromatic hydrocarbons (PAHs) were selected as the target analytes. Under the optimum extraction and desorption conditions, the limit of detection was as low as 3.0 ng L^−1^, and the linear range was 0.01–20.0 μg L^−1^. The enrichment factors of PAHs were in the range of 1724–2393. Three environmental water samples of mineral water, tap water and river water were analyzed by this method, and the recoveries that spiked at 1.0–10.0 μg L^−1^ were between 80.5–126%. It showed many advantages compared with other methods, such as better sensitivity, faster detection and online analysis.

## 1. Introduction

Solid-phase microextraction (SPME), as a simple and efficient method, has been widely used in recent years [1]. Compared with liquid-phase extraction, solid-phase extraction and other traditional advantages [2], in-tube SPME is performed with no organic solvent and less sample, and it can further be combined with high-performance liquid chromatography (HPLC) for online analysis and better accuracy [3,4]. The selection of extraction materials is particularly important for SPME [5,6]. Various types of extraction materials such as organic polymers [7,8], covalent organic frameworks [9,10], metal-organic frameworks [11,12] and carbon materials [13,14] all have large surface areas and many adsorption sites or functional groups, and these properties are the necessary conditions of effective extraction materials [15,16,17].

Aerogels have been widely used in construction, aerospace, environmental protection and catalysis due to their extremely low density, high specific surface area, high porosity and easy preparation [18,19]. Silica aerogel, as the earliest aerogel material, not only possesses all the advantages of aerogels, but also has a mature and cheap synthesis method [20]. Although silica aerogels as extraction materials displayed many advantages, their adsorption performance for hydrophobic analytes is unsatisfactory due to a large amount of hydrophilic hydroxyl groups on its surface [21,22]. Melamine-terephthalaldehyde (MT) polymer is an organic polymer composed of C, H, O and N elements, with excellent chemical and thermal stability. Due to rich functional groups such as amino, triazinyl, phenyl, ether and alkyl groups in this polymer, multi-interactions including π-π and hydrophobic effect can produce. In order to combine the advantages of the two materials, the silica aerogel can be hybridized by the melamine-terephthalaldehyde polymer, the obtained composite has both a high specific surface area and many adsorption sites. Based on these considerations, this work adopted the sol–gel method to produce a melamine-terephthalaldehyde polymer-hybridized silica (MT-SiO_2_) aerogel. The stainless steel wires were coated by the aerogel, and placed into a polyetheretherketone (PEEK) tube to prepare an extraction tube. Polycyclic aromatic hydrocarbons (PAHs) are one type of important hydrophobic organic pollutants in the environment. Several PAHs were selected as the model analytes to evaluate the extraction tube. After connecting the tube with HPLC, the effect of the extraction and desorption conditions on the extraction efficiency was carefully investigated. The online analytical method was established and applied to the analysis of actual samples.

## 2. Materials and Methods

### 2.1. Materials and Reagents

Polyetheretherketone tube (0.75 mm i.d., 1.6 mm o.d.) was obtained from Changzhou Youwoshi Plastic Products Co., Ltd. (Changzhou, China). Stainless steel wire (0.18 mm d.) was made by Jiangsu Yixing Shenglong Wire Mesh Co., Ltd. (Yixing, China). Ammonia (28%) was an analytical reagent from Laiyang Economic and Technological Development Zone Chemical Factory (Laiyang, China). Dimethyl sulfoxide (DMSO), naphthalene (Nap), acenaphthylene (Acy), acenaphthene (Ace), fluorene (Flu), phenanthrene (Phe), anthracene (Ant), fluoranthene (Flt) and pyrene (Pyr) were all analytical reagents from Shanghai Aladdin Bio-Chem Technology Co., Ltd. (Shanghai, China). Melamine, terephthalaldehyde and ethylorthosilicate were purchased from J&K Scientific Ltd. (Beijing, China). Methanol and acetonitrile were of chromatographic grade from Tedia Chemical Reagent Co. (Fairfield, CT, USA). Hydrochloric acid (12 mol L^−1^) was an analytical reagent from Tianjin Fuyu Chemicals Co., Ltd. (Tianjin, China). Ultrapure water was used for all tests.

### 2.2. Apparatus

An Agilent 1260 HPLC system (Agilent Technologies, Santa Clara, CA, USA) with a Zorbax C18 column (250 × 4.6 mm i.d., 5 µm) and a diode array detector (DAD) was applied in the experiment. Gradient elution (0–10 min, acetonitrile-water (70:30, *v*/*v*), 10–20 min, acetonitrile increased to 100%) was performed for detecting PAHs. All detection was performed under 1.00 mL min^−1^ of flow rate and 25 °C of column temperature. A P1201 pump was purchased from Dalian Elite Analytical Instrument Co., Ltd. (Dalian, China), used to carry the sample solution. A scanning electron microscope (SEM, Supratm55, Carl Zeiss, AG, Germany) was used to survey MT-SiO_2_ aerogel.

### 2.3. Preparation of Extraction Tube

**Figure 1:**
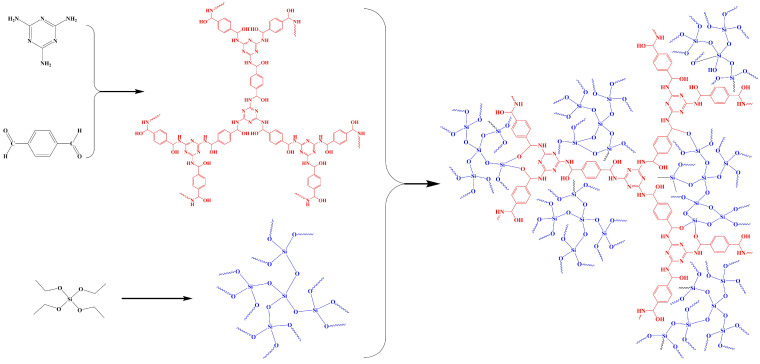
Preparation mechanism of MT-SiO_2_ aerogel.

The preparation schematic of MT-SiO_2_ aerogel is shown in Figure 1. Firstly, melamine of 0.6260 g and terephthalaldehyde of 1.0000 g were dissolved into 31 mL of DMSO. Then, the solution was heated to 150 °C under the protection of argon for polymerization, after 48 h it was cooled to room temperature. The obtained precipitation was washed with acetone, tetrahydrofuran and dichloromethane three times in turn, then the product was dried in a vacuum oven at 65 °C to get the MT polymer.

Secondly, 0.0100 g, 0.0200 g, 0.0300 g, 0.0400 g and 0.0500 g MT polymers were, respectively, added into 3 mL ethanol and dispersed by ultrasound. Then, 1 mL of ethylorthosilicate was added and stirred. After 15 min, 1 mL of ultrapure water was added. After 30 min, 10 μL of 0.1 mol L^−1^ hydrochloric acid was added to adjust solution pH to 3–4. After the solution was heated at 50 °C for 8 h, it was cooled to room temperature, ammonia solutions (7%) of 15 μL, 20 μL, 30 μL, 40 μL and 45 μL were separately added into the above five reaction solutions to adjust pH to 7–8, making it react to form a gel. The solvent of the gel was exchanged with ethanol through immersing the gel for 5 h each time and ten times, then the gel was dried at 80 °C for 6 h, and the MT-SiO_2_ aerogel was obtained.

Lastly, two extraction tubes were prepared. MT-SiO_2_ aerogel and silica aerogel powders were obtained by grinding. They were glued on a 40 cm stainless steel wire with epoxyresin and the coating was dried for 48 h at room temperature. Put four aerogel-coated wires in a 30 cm PEEK tube, cut off the excessive end of the wires and flushed with 1.00 mL min^−1^ of water and ethanol for 30 min by HPLC pump.

### 2.4. Online Analytical Procedure

The specific method of in-tube SPME combined with HPLC online can be seen in our previous research [23]. The extraction and desorption steps of the analytes would be changed by revolving the six-way valve of HPLC. When the valve was in the load state, the sample solution containing the analytes flowed through the extraction tube under the push of the pump to complete the extraction process. After extraction, the valve was switched to the inject state, the mobile phase of HPLC flowed through the tube to desorb the extracted analytes. The analytes were sent to the HPLC column for chromatographic separation and detection.

### 2.5. Sample Preparation

A stocking solution of eight PAHs (Nap, Acy, Ace, Flu, Phe, Ant, Flt, Pyr) was prepared to 10.0 mg L^−1^ in methanol and was stored at 4 °C. Working solutions were prepared by diluting the stocking solution to a required concentration in ultrapure water. Mineral water, tap water and lake water were selected as actual samples, which were filtered before testing.

## 3. Results

### 3.1. Characterization

**Figure 2:**
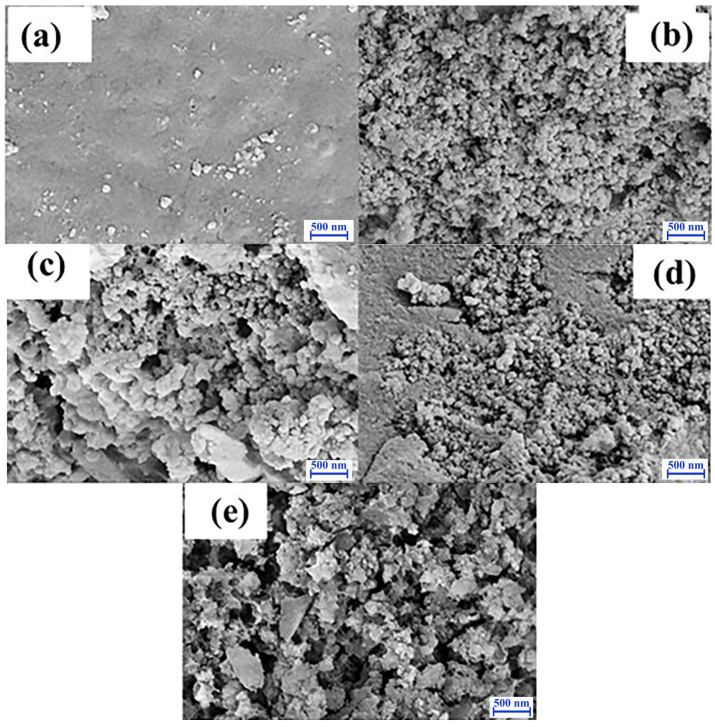
SEM images of MT-SiO_2_ aerogels with different contents of MT polymer. MT polymer content: (**a**) 0.0100 g, (**b**) 0.0200 g, (**c**) 0.0300 g, (**d**) 0.0400 g and (**e**) 0.0500 g.

The MT-SiO_2_ aerogels with different contents of MT polymer were surveyed by SEM. As shown in Figure 2a, when the MT polymer content was 0.0100 g, the material surface was compact and smooth. In Figure 2b–e, with the increase in MT polymer from 0.0200 g to 0.0500 g, the surface of these materials became loose and porous. Larger surface area provides more adsorption sites, but aerogel could not be prepared when the MT polymer content was more than 0.0500 g. Therefore, the following experiment was carried out with MT-SiO_2_ aerogel containing 0.0500 g MT polymer.

### 3.2. Investigation of Extraction and Desorption Conditions

**Figure 3:**
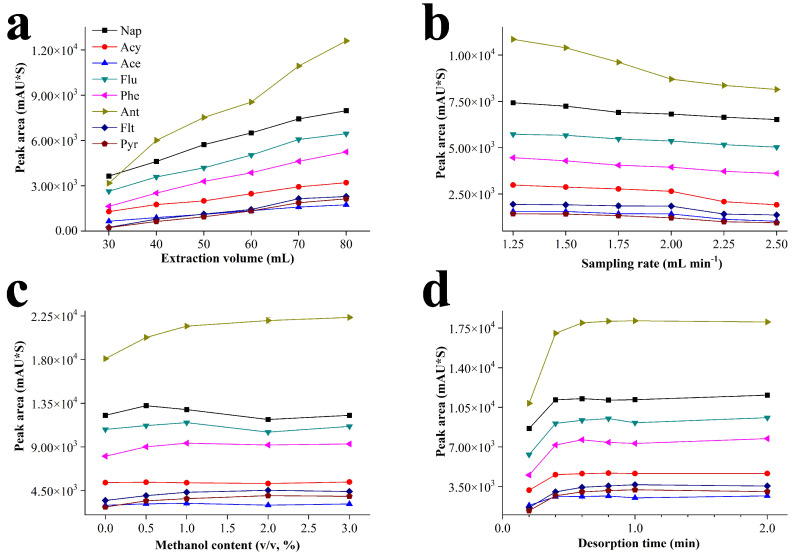
The effect results of (**a**) extraction volume, (**b**) sampling rate, (**c**) methanol content and (**d**) desorption time.

In order to get the best test results, the extraction volume, sampling rate, organic solvent content and desorption time were optimized. The working solution of PAHs was set as 5.0 μg L^−1^. In general, the extraction efficiency increases by enlarging the sampling volume or reducing the sampling rate, but the test time is extended. The optimization of extraction volume and sampling rate is to find a balance between extraction efficiency and working efficiency. Sample solution of 30–80 mL and sampling rate of 1.25–2.50 mL min^−1^ were selected for the optimization. As shown in Figure 3a, when the extraction volume was enlarged from 30 to 80 mL, the peak areas of all substances are grown gradually. In Figure 3b, the peak area of each target slowly declined from 1.25 to 2.00 mL min^−1^, the peak areas of Acy, Flt, Ace and Pyr were further decreased greatly when the sampling rate exceeded 2.00 mL min^−1^. In order to achieve satisfactory extraction efficiency and test time, 70 mL and 2.00 mL min^−1^ were selected as the best extraction volume and sampling rate, respectively.

Adding an appropriate amount of organic solvent in the sample solution increases the solubility of hydrophobic analyte in water, obtaining good test repeatability and extraction effect. Too much organic solvent reduces the distribution coefficient of PAHs between the extraction coating and sample solution. In this experiment, methanol was selected as an organic solvent to be added in the sample solution, and the concentration was controlled as 0, 0.5%, 1.0%, 2.0% and 3.0% (*v*/*v*), respectively. As shown in Figure 3c, except for Acy, other analytes presented a slight increase with methanol from 0 to 1.0% (*v*/*v*). When the methanol was more than 1.0% (*v*/*v*), little change was seen for all peak areas. Lastly, 1.0% (*v*/*v*) of methanol was selected in the next tests.

The desorption efficiency of the analyte from the extraction tube significantly affects the analytical results. If the desorption time is insufficient, not only the analytical result is inaccurate, but the residual target in the tube also influences the next test. The desorption time was carefully investigated from 0.2 min to 2.0 min, the results are summarized in Figure 3d. According to the increased tend of all peak areas, the desorption of the target was incomplete when the time was less than 0.6 min. In order to obtain accurate results and eliminate the influence of residual, 2.0 min was selected as the optimal desorption time.

In summary, the optimized conditions were as follows: the extraction volume was 70 mL, sampling rate was 2.00 mL min^−1^, methanol concentration in the sample was 1.0% (*v*/*v*) and the desorption time was 2.0 min.

### 3.3. Method Evaluation and Sample Analysis

This in-tube SPME-HPLC method was evaluated by testing a series of standard solutions of PAHs under optimized conditions. As can be seen from the results in Table 1, the method had wide linear ranges, low limits of detection (LODs, three times the signal-to-noise ratio), high EFs and satisfied repeatability for eight PAHs. The linear range was 0.016–10.0 μg L^−1^ for Phe, Flt and Pyr, it was 0.016–20.0 μg L^−1^ for Ace and Ant and it was 0.010–15.0 μg L^−1^, 0.010–20.0 μg L^−1^ and 0.016–15.0 μg L^−1^ for Nap, Flu and Acy, respectively. Low LODs in 3.0–5.0 ng L^−1^ resulted from the high enrichment effect of the tube, and EFs of eight PAHs were 1724–2393. In addition, the repeatability of the method was investigated by intra-day and inter-day tests, and the RSD (*n* = 5) of each analyte ranged from 0.61 to 8.3% and 6.8 to 18%, respectively.

**Figure 4:**
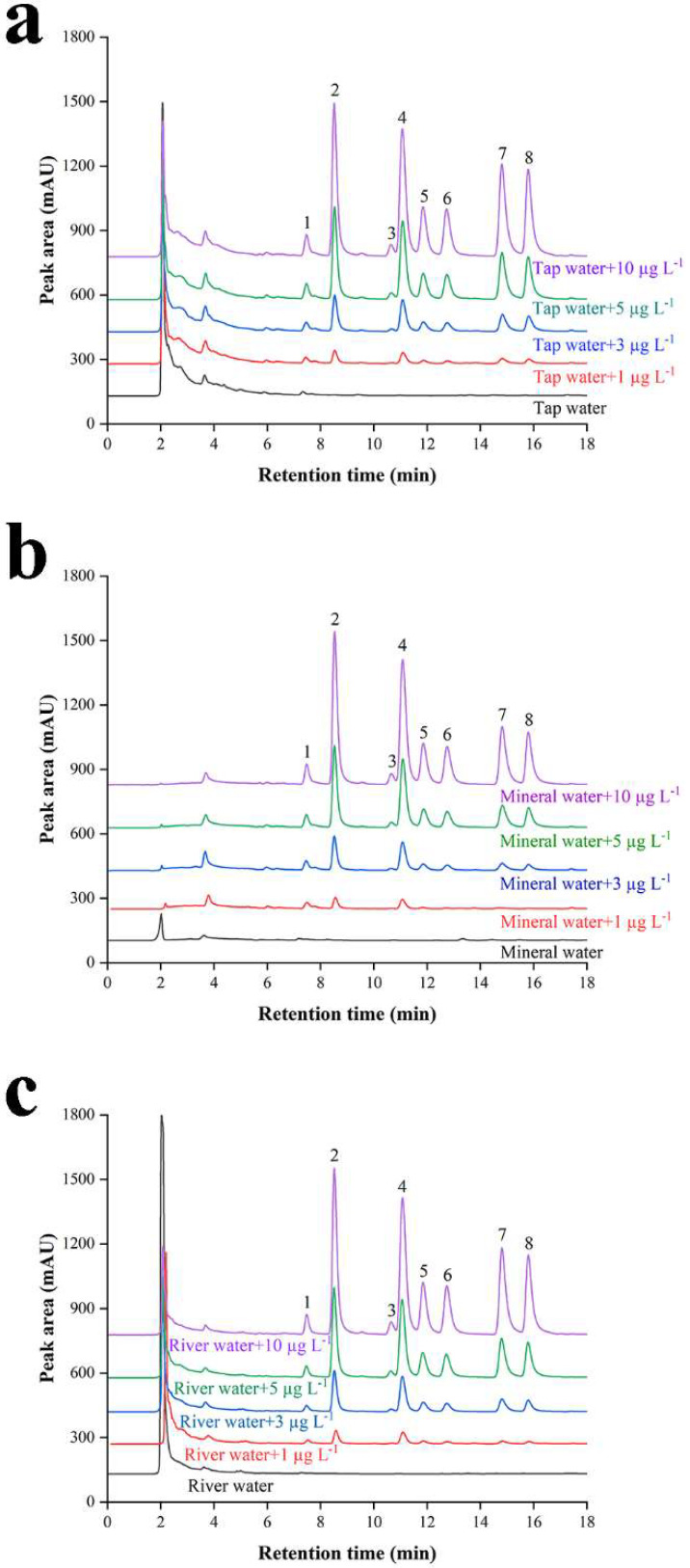
The chromatograms of real water samples including (**a**) tap water, (**b**) mineral water and (**c**) river water. Analytes: (1) Nap, (2) Acy, (3) Ace, (4) Flu, (5) Phe, (6) Ant, (7) Flt and (8) Pyr.

The PAHs in tap water, mineral water and river water were detected by this method, and the recoveries were determined by adding different levels including 1.0 μg L^−1^, 3.0 μg L^−1^, 5.0 μg L^−1^ and 10.0 μg L^−1^. The results are shown in Table 2 and Figure 4, no target was detected in tap water and mineral water, and there was a small amount of Nap in river water, but the content could not to be quantified. In these samples, the relative recovery of each analyte spiked from 1.0 to 10.0 μg L^−1^, ranged from 80.5 to 126%. It was proved that the method could be applied to actual sample detection.

### 3.4. Comparison with Other Methods

The established method was compared to other analytical methods based on other extraction materials. According to the results in Table 3, compared with the fiber SPME-HPLC-UVD [24] and SBSE-HPLC-UVD methods [25], this method achieved lower LODs, and although the linear range was not larger, it saved much more time. In the similar extraction time, the LODs were lower than that from the fiber SPME-HPLC-UVD method [26]. Furthermore, the method was online extraction and detection, which could give better repeatability than these offline methods. Compared with some online methods including in-tube SPME-HPLC-FLD [27] and in-tube SPME-HPLC-DAD [28,29], this method also had some advantages such as lower LODs and larger or comparable linear ranges. Better results of the proposed method were attributed to the superior extraction property of MT-SiO_2_ aerogel over some extraction materials. In addition, three extraction tubes were produced, their extraction performance under the same conditions was compared with each other. The RSD results of analyte peak areas among the three tubes were less than 23%, which is acceptable. After 100 runs on one extraction tube, the extraction efficiency still remained more than 85% for each analyte, proving the satisfactory stability during the whole test.

## 4. Conclusions

In this paper, a new polymer-hybridized silica aerogel was developed for in-tube SPME. The aerogel was coated on stainless steel wire, then several wires were placed into a polyetheretherketone tube to get an extraction tube. Coupled with HPLC, the tube was evaluated by several PAHs as target analytes. After the extraction and desorption conditions were optimized, an online analytical method was developed for determining trace PAHs in water samples, with enrichment effect up to 2393 times and LOD as low as 3.0 ng L^−1^ in a 35 min test time. This method successfully determined trace target from environmental water samples. This research not only enriches aerogel materials but also provides a reference for future research on extraction, detection and analysis of environmental pollutants.

## Figures and Tables

**Table 1:** The results of the method evaluation.

Analytes	Linear Ranges (μg L^−1^)	LODs (ng L^−1^)	Linear Coefficients	EFs ^a^	Repeatability (*n* = 5, RSD%)	
					Intra-Day	Inter-Day
Nap	0.010–15.0	3.0	0.9962	2055	0.61	7.0
Acy	0.016–20.0	5.0	0.9997	2061	1.5	6.8
Ace	0.016–15.0	5.0	0.9997	2393	1.3	15
Flu	0.010–20.0	3.0	0.9990	2289	2.3	12
Phe	0.016–10.0	5.0	0.9989	2271	3.1	18
Ant	0.016–20.0	5.0	0.9993	1951	3.3	12
Flt	0.016–10.0	5.0	0.9937	2052	8.3	12
Pyr	0.016–10.0	5.0	0.9920	1724	8.2	9.5

^a^ EF = C_SPME_/C_0_, 5.00 μg L^−1^ (C_0_) of sample was tested, and the corresponding C_SPME_ with same peak area was obtained by direct injection of 20 μL concentrated samples.

**Table 2:** Analytical results and relative recoveries of several PAHs in real water samples.

Real Samples	Analytes	Detection Results	Recovery (%) ^a^	Recovery (%) ^b^	Recovery (%) ^c^	Recovery (%) ^d^
Tap water	Nap	ND	100	84.4	119	97.2
	Acy	ND	83.8	96.6	102	101
	Ace	ND	84.4	81.9	113	112
	Flu	ND	87.2	80.5	112	106
	Phe	ND	89.4	89.3	116	126
	Ant	ND	99.8	89.2	117	121
	Flt	ND	102	96.9	118	118
	Pyr	ND	118	111	117	112
Mineral water	Nap	ND	99.3	83.8	111	93.4
	Acy	ND	87.9	93.0	97.6	94.1
	Ace	ND	98.9	91.4	95.0	97.9
	Flu	ND	83.0	92.5	99.9	96.1
	Phe	ND	83.2	87.8	100	105
	Ant	ND	86.1	86.9	103	106
	Flt	ND	87.8	81.9	93.1	107
	Pyr	ND	96.5	99.6	95.9	111
River water	Nap	NQ	85.7	83.3	112	95.6
	Acy	ND	85.6	89.7	107	91.8
	Ace	ND	80.8	84.6	117	104
	Flu	ND	90.0	93.1	116	98.3
	Phe	ND	97.5	86.6	114	121
	Ant	ND	90.4	94.8	119	104
	Flt	ND	121	114	106	113
	Pyr	ND	116	121	102	114

ND, not detected. NQ, not quantified. ^a^ Standard addition level at 1.0 μg L^−1^. ^b^ Standard addition level at 3.0 μg L^−1^. ^c^ Standard addition level at 5.0 μg L^−1^. ^d^ Standard addition level at 10.0 μg L^−1^.

**Table 3:** Comparison of the analytical method with other methods for the determination of PAHs.

Methods	Extraction Materials	LODs (ng L^−1^)	Linear Ranges (μg L^−1^)	Extraction Time (min)	Analytical Mode	References
In-tube SPME-HPLC-DAD	MT-SiO_2_ aerogel	3.0–5.0	0.01–20	35	Online	This work
Fiber SPME-HPLC-UVD	C_12_-Ag wire	580–1860	5–200	60	Offline	[24]
SBSE-HPLC-MS/MS	Polydimethylsiloxane	1–22	0.01–100	180	Offline	[25]
Fiber SPME-HPLC-UVD	Multiwall carbon nanotube/ZrO_2_	33–160	0.1–200	30	Offline	[26]
In-tube SPME-HPLC-FLD	Zeolitic imidazolate framework-8 polydopamine	5–50	0.01–5	25	Online	[27]
In-tube SPME-HPLC-DAD	Mesoporous titanium oxide	10–100	0.03–30	36	Online	[28]
In-tube SPME-HPLC-DAD	Nano-calcium carbonate	50–300	0.15–20	26	Online	[29]

## Data Availability

Data are available from the authors.
